# Congenital Cytomegalovirus Infection in Malaysia: A Scoping Review, Clinical Insights and Future Directions

**DOI:** 10.21315/mjms-05-2025-334

**Published:** 2025-08-30

**Authors:** Asrar Abu Bakar, Wan Muhammad Najib Wan Mahmud Sabri, Nurlin Ali Hanafiah, Nurfarisha Ahmad Fazli, Ciang Sang Tan, Haema Shunmugarajoo, Nik Khairulddin Nik Yusoff

**Affiliations:** 1 Department of Paediatrics, International Islamic University Malaysia, Kuantan, Pahang, Malaysia; 2 Department of Paediatrics, Sultan Ahmad Shah Medical Centre @IIUM, International Islamic University Malaysia, Kuantan, Pahang, Malaysia; 3 Department of Audiology and Speech-Language Pathology, International Islamic University Malaysia, Kuantan, Pahang, Malaysia; 4 Faculty of Medicine, Universiti Malaya, Kuala Lumpur, Malaysia; 5 Department of Paediatrics, Malacca General Hospital, Melaka, Malaysia; 6 Department of Paediatrics, Hospital Raja Permaisuri Bainun, Ipoh, Perak, Malaysia; 7 Departnent of Paediatrics, Hospital Raja Perempuan Zainab II, Kota Bharu, Kelantan, Malaysia

**Keywords:** cytomegalovirus, congenital, neonates, infection, neurodevelopment

## Abstract

Congenital cytomegalovirus (cCMV) infection is the most common congenital infection worldwide, greatly contributing to neonatal morbidity and long-term neurological and audiological complications. Despite its clinical burden, cCMV remains underdiagnosed in Malaysia due to the absence of routine screening programmes and limited awareness among healthcare professionals. This review presents an overview of the epidemiology, clinical manifestations, diagnostic approaches, and management strategies of cCMV in Malaysia, highlighting gaps in current practices and potential future approaches. The seroprevalence of cytomegalovirus (CMV) among pregnant women in Malaysia is high, with reported maternal seropositivity rates between 74% and 84%. However, cCMV incidence remains poorly defined due to the lack of systematic screening. Clinical features range from asymptomatic infection to severe neurodevelopmental impairments, sensorineural hearing loss, and visual deficits. Current diagnostic methods rely on polymerase chain reaction (PCR)-based detection of CMV DNA from urine, saliva, or blood within the first three weeks of life. However, access to molecular testing is limited. Treatment with antiviral agents such as valganciclovir can improve neurodevelopmental outcomes, particularly in symptomatic infants, but remains underutilised due to cost and availability constraints. Emerging research on rapid antigen-based diagnostics and maternal antiviral prophylaxis offers opportunities for improving early detection and intervention. Future strategies should focus on increasing awareness among healthcare providers, implementing targeted or universal newborn screening, and promoting research on cost-effective diagnostic and treatment modalities. Strengthening national policies and integrating cCMV management into existing maternal and child health programmes is vital to mitigate its long-term impact.

## Introduction

Cytomegalovirus (CMV) is a common DNA herpesvirus that readily infects healthy and immunocompromised individuals. Globally, it carries a substantial health burden, with seroprevalence of up to 83% in the general population and 86% in women of reproductive age (1). Immunocompetent individuals, including pregnant women, can control the virus, rendering them asymptomatic or with mild symptoms upon infection (2). However, CMV can cause debilitating symptoms among newborns infected by seropositive mothers and can lead to neurocognitive impairment and deafness (3–4). Antenatal screening for CMV remains inconsistent and limited, particularly in low-income countries, while efforts continue to develop vaccines (5).

Congenital CMV (cCMV) is defined by evidence of CMV infection demonstrated by DNA detection from the newborn’s urine, saliva, or blood during the first three weeks of life; infection beyond three weeks is likely acquired postnatally. cCMV is the most common congenital infection globally, with an estimated prevalence of 0.2% to 2% in all live births (6). It is second only to cerebral palsy as a leading cause of developmental malformations in many parts of the world (7). Despite its recognised burden, existing literature regarding prevalence, disease profile, outcomes, and treatment strategies for cCMV in Malaysia is limited. Literature searches on PubMed, Scopus, Embase, and Google Scholar identify 12 articles relevant to cCMV or seropositive childbearing women in Malaysia, with the oldest paper dating back to 1976.

## Epidemiology of cCMV

Overall, the risk of CMV transmission from a seropositive mother to her newborn can reach up to 50% in primary infections but is significantly lower in reinfections (< 2%) (8). Primary infection is associated with higher cytokine expression, resulting in a more active viral state and increased transmissibility (9). Maternal infection acquired during the first trimester has shown lower transmissibility compared to higher transmission rates in third-trimester infections (10). However, first trimester infections are linked with more severe end-organ damage due to the virus’s neurotropic effect on a developing fetus (11).

The prevalence of cCMV correlates with maternal seropositivity, with incidence rates being 3–4 times higher in lower-income countries where CMV prevalence is elevated (12–13). Random sampling of healthy pregnant women across various hospitals in Malaysia revealed a CMV seropositivity rate of 74% to 84%, with CMV IgM positivity, indicating recent infection or reinfection, detected in 7% of cases (14–15). Maternal seropositivity was consistently distributed among major racial groups, including Malays, Chinese, and Indians (16).

Among newborns, seropositivity rates were as high as 94% to 100% at birth, reflecting strong maternal immunity. However, these levels declined to approximately 50% to 70% by four months of age, likely due to the waning of maternal antibodies (17). In a Malaysian cross-sectional survey of 1,688 infants with congenital anomalies, 11% tested positive for CMV IgM antibodies, a prevalence higher than other congenital infections (18). Among 648 infants screened for symptomatic congenital infections, only 0.3% met the diagnostic criteria for cCMV, although early molecular testing was not widely implemented (15).

Meanwhile, the prevalence of premature infants with cCMV remains largely unknown. Few observational studies estimate a range between 3% to 16.7% globally, while data in Malaysia is lacking (19–20). Premature infants have a higher rate of symptomatic disease and increased risk of developing hearing impairment and neurodevelopmental disorders compared to their term counterparts (21–22). However, the lack of routine testing for CMV complicates the estimation of disease risk and burden in this unique yet essential population.

## Clinical Features of cCMV

Globally, the majority of infants (85% to 90%) infected with cCMV remain asymptomatic, a pattern that is also observed among cCMV-infected infants in Malaysia (15). While fetal infection during the first trimester presents a lower risk of vertical transmission, it is associated with a higher likelihood of early symptomatic disease and clinical sequelae (23). However, symptomatic disease may be overlooked in infants with silent infection, where end-organ involvement is only detectable through screening and clinical evaluations. In high-income settings, the recognition of symptomatic cases has improved due to targeted screening and advances in neuroimaging techniques such as MRI. Consequently, the distinction between asymptomatic and symptomatic or clinically apparent disease is not as clear-cut as previously assumed, especially in low-income settings where screening is limited (24).

### Systemic Features

The clinical presentation of cCMV varies according to the extent of end-organ involvement and includes physical features, laboratory abnormalities, neuroimaging findings, as well as visual and auditory impairments. Symptoms can range from a single clinical symptom to widespread disease affecting multiple organs. At birth, clinically apparent manifestations may include small for gestational age, prematurity, microcephaly, hepatosplenomegaly, prolonged jaundice, and skin abnormalities such as blueberry muffin rash, petechiae, or purpura (7). Laboratory abnormalities may involve haematological anomalies, including unexplained anaemia, neutropenia, thrombocytopenia, and elevated liver enzymes and conjugated bilirubin levels (7). In Malaysia, jaundice was the most commonly observed clinical finding among acutely symptomatic infants (15).

### Central Nervous System Features

Central nervous system (CNS) manifestations of cCMV include poor muscle tone, poor suck reflex, and seizures detected at birth. Nonetheless, CNS presentations are often subclinical and depend on imaging to thoroughly evaluate neurological involvement. Typical neuroimaging findings include calcifications, periventricular cysts with or without ventricular dilatation, lenticulostriate vasculopathy, subependymal pseudocysts, and cortical atrophy, which are often better observed using MRI (25).

White matter abnormalities, on the other hand, have been associated with moderate to severe disability and hearing loss. In contrast, other studies appear more guarded and reflect difficulties in interpreting pathological changes in a maturing brain (26–27). Nonetheless, long-term CNS complications can range from severe cognitive and motor impairments, such as cerebral palsy, to other socio-developmental disorders, including autism and speech or language delays (25). It remains uncertain which CNS features at birth predict these outcomes, and ongoing research examines these associations.

### Audiological and Ophthalmological Features

cCMV is the leading non-genetic cause of hearing loss in children, affecting 40% to 50% of symptomatic infants at birth and 7% to 10% of asymptomatic cases (28–29). Audiological complications generally present as sensorineural hearing loss, which may be unilateral or bilateral and can fluctuate or worsen over time (30). Visual impairments occur in 5% to 30% of cCMV-infected infants, potentially involving all segments of the eye and visual pathways (31–32). Early manifestations include chorioretinitis, strabismus, amblyopia, and cataracts, while late complications may involve optic nerve atrophy and central visual impairment (32).

### Disease Category

Due to the variability in clinical presentation and symptom heterogeneity, assessing the severity of symptomatic cCMV disease remains difficult. Expert consensus categorises cCMV disease into mild-moderate or severe forms, with mild or moderate disease defined as isolated, clinically insignificant, or temporary findings such as petechiae, mild hepatomegaly or splenomegaly, or self-limiting biochemical and haematologic abnormalities (7, 25). In contrast, severe disease is characterised by life-threatening manifestations, any CNS involvement, chorioretinitis, or early onset sensorineural deafness (7, 25). These classifications are essential for guiding clinical decisions regarding antiviral treatment initiation.

Nonetheless, different centres have varying agreements on the definition of disease severity, specifically in the context of isolated mild hearing impairment or isolated abnormal white matter changes seen on MRI in a similarly well-appearing, clinically asymptomatic infant (33). Hence, treatment decisions differ between centres for these patients. Much remains to be understood, particularly regarding abnormal neurological findings at diagnosis, the extent of these abnormalities, and their implications for future neurodevelopmental outcomes. Long-term follow-up studies are necessary, as learning difficulties and social communication deficits may only become apparent around the fourth year of life and beyond (34). Establishing these relationships is vital to allow a much clearer definition of symptomatic disease requiring treatment.

## Screening and Diagnosis of cCMV

### Antenatal Screening

Routine screening for maternal CMV serostatus during pregnancy is not commonly conducted in Malaysia. However, advancements in ultrasound imaging and increased awareness of its interpretation have enabled more targeted antenatal screening. Pregnant women presenting with influenza-like illness, glandular fever, or hepatitis have also been identified for focused CMV screening (35). The gold standard for diagnosing fetal CMV infection is CMV polymerase chain reaction (PCR) on amniotic fluid, with test reliability increasing when amniocentesis is performed at least eight weeks after primary maternal infection (36).

If amniocentesis is not possible, maternal CMV serology can help estimate the risk and guide postnatal testing. However, negative serology or the presence of CMV IgG alone does not reliably exclude fetal infection (37). Adding an IgG avidity test which measures the strength of antigenic binding helps differentiate between a primary infection during pregnancy or immunity from previous infection (38). Higher avidity during the first trimester indicates a higher probability of infection acquired in the periconceptional or preconceptual period. In contrast, lower avidity at later trimesters indicates a higher risk of mother-to-fetus transmission (10). Despite these methods, most CMV screenings occur postnatally, often following evaluations for other conditions such as prolonged jaundice, which can lead to delays in cCMV diagnosis and treatment. Antenatal screening thus provides an opportunity to identify affected infants earlier.

### Postnatal Screening: Universal and Targeted

Since the majority of infants born with cCMV are asymptomatic and symptoms might be subclinical, delayed diagnosis and treatment are inevitable without a universal screening programme (39). Established universal programmes most frequently use dried blood spot (DBS) samples to identify potentially infected newborns, with additional protocols initiated upon detection, including confirmatory urine or saliva viral load testing. Such programmes would benefit from time-sensitive antiviral treatment and prevent neurocognitive and audiological complications.

Nonetheless, lack of evidence on the cost-effectiveness of universal screening has limited its implementation. It is currently practised in only a few countries, such as some provinces in Canada, and several states in the United States (40). Many institutions have adopted a targeted screening approach and screen infants who are symptomatic, at-risk groups such as premature infants, and those who failed routine newborn hearing screening. This allows a more focused diagnostic approach to detect those who are symptomatic and would likely benefit from treatment. In Malaysia, postnatal screening has not been established and is typically performed only in suspicious cases (15).

### Confirmatory Test

In the absence of routine screening programmes, infants are often diagnosed with cCMV postnatally, typically in those who have developed symptoms. Early and accurate diagnosis is essential, as delayed intervention can have serious consequences. The gold standard for diagnosing cCMV is the detection of CMV DNA in urine, saliva, or blood within the first three weeks of life, with quantitative polymerase chain reaction (qPCR) being the preferred technique due to its high sensitivity and specificity (41). Urine PCR offers greater sensitivity than saliva PCR, while blood PCR may produce negative results in up to 25% of cCMV cases due to the absence of viremia (42). In Malaysia, qPCR for CMV is primarily conducted in centralised laboratory units, making the process labour-intensive and expensive, limiting access to early diagnosis.

Postnatal serological assays, such as CMV IgM and IgG taken from infants, require careful interpretation due to the virus’s latent and reactivation properties. Yet, they are often used in low-resource settings because of greater availability and low cost. Serology tests, however, should not be used as diagnostic confirmation as acquired maternal antibodies can persist beyond a few months to years following infection, and false positives may result from sera cross-reactivity (43). It has been suggested that a paired serum sample taken 2–4 weeks apart would increase the likelihood of an acute CMV infection; however, limitations still exist given low sensitivity (44–45). Nonetheless, a negative CMV IgG at birth is a valuable test to exclude cCMV (37).

### Imaging

Cranial imaging plays a crucial role in detecting CNS involvement and supporting the diagnosis of cCMV; therefore, it should be considered a routine evaluation for all suspected cases. Cranial ultrasound is the first-line imaging method, with CNS abnormalities including lenticulostriate vasculopathy (LSV), periventricular calcifications, cysts, and ventriculomegaly (37). However, MRI is increasingly emphasised, as it can identify abnormalities such as white matter changes and cortical malformations that are not apparent on ultrasound (46). Isolated cerebellar hypoplasia, detectable via MRI, has also been reported (47). More importantly, abnormalities seen on MRI were detected in up to 33% of clinically asymptomatic infants (48).

Additionally, the extent of white matter involvement is a valuable prognostic indicator for neurodevelopmental outcomes, with temporal pole abnormalities associated with an increased risk of hearing loss (26). Infants with extensive polymicrogyria are at a higher risk of developing epilepsy later in life (49). As a result, a combination of cranial ultrasound and MRI is recommended to improve the detection of symptomatic infants who may benefit from treatment, with emerging evidence supporting its role as a prognostic tool. Nonetheless, the availability of MRI might be limited in many healthcare institutions in Malaysia. Therefore, a practical approach would be to perform MRI on at-risk groups such as premature or low birthweight infants and those with symptomatic disease, including isolated transaminitis or haematological abnormalities.

## Treatment of cCMV

### Antiviral Indication and Duration

The treatment approach for cCMV has advanced, with antiviral therapy serving as the primary intervention for symptomatic infants. Ganciclovir and its prodrug, valganciclovir, are approved by the National Pharmaceutical Regulatory Agency; however, their use for cCMV remains off-label. They have been shown to prevent hearing deterioration, particularly when administered early in neonates with symptomatic cCMV (50). A six-month regimen of valganciclovir has been shown to reduce viral loads while modestly improving both hearing and neurodevelopmental outcomes (51). This regimen is now considered the standard of care for infants with symptomatic congenital CMV at birth, particularly those who failed newborn hearing screening, with chorioretinitis or notable intracranial abnormalities (7, 37).

It is usually reasonable to monitor cases of mild to moderate disease, such as isolated persistent transaminitis, anaemia, or thrombocytopenia, as these conditions are often temporary and resolve spontaneously. However, in infants with persistent symptoms beyond two weeks without improvement, a shortened six-week course of treatment may be considered (37). There is no strong evidence supporting treatment for isolated intrauterine growth restriction. The decision to initiate therapy must carefully weigh the potential risks of drug toxicity, requiring close monitoring of haematologic and renal parameters. Nevertheless, longer longitudinal studies are needed to better understand the impact of treatment versus non-treatment in infants with mild to moderate symptoms, as complications, including sensorineural deafness, can appear at a later stage.

The timing of treatment initiation has also been shown to significantly influence outcomes in symptomatic cCMV infants. Randomised trials have linked treatment within the first 21 days of life to improved outcomes (50). However, delays in treatment often occur due to limited access to molecular testing, such as qPCR, which is essential for early diagnosis. Oral valganciclovir therapy started beyond one month has not been shown to improve hearing outcomes (52).

There is no consensus on whether viral load monitoring is necessary to guide treatment duration in addition to clinical assessments. A reduction of both blood and urine CMV viral loads would indicate a favourable treatment response, and failure to achieve a reduction might signify viral resistance. Nonetheless, while higher viral loads are associated with more severe forms of cCMV at the time of diagnosis, there is limited evidence of its influence on long-term outcomes (53–54). Therefore, regular viral load monitoring and documented evidence of viral clearance are yet to be recommended as routine standards of care.

### Dosing Strategies

There are no validated pharmacokinetic-pharmacodynamic (PK/PD) trials conducted in neonates for ganciclovir or valganciclovir. The dosing regimen of 6 mg/kg twice daily for intravenous ganciclovir was established from small trials and remains the recommended dose for term infants (55). Similarly, the studied dose for its oral prodrug, valganciclovir, is 16 mg/kg twice daily, producing plasma concentrations comparable to the intravenous formulation (56). These dosing recommendations are appropriate in infants born beyond 32 weeks of gestation and weighing more than 1.8 kg (56). As the kidneys primarily excrete these drugs, careful monitoring is essential for infants born prematurely or with very low birth weights, as these doses have been linked to increased plasma levels and potential toxicity (57). Further PK/PD studies are required to determine the appropriate dosing for preterm infants diagnosed with cCMV.

### Side Effects and Therapeutic Drug Monitoring

It is essential that all infants started on therapy be closely monitored for bone marrow suppression, including anaemia, neutropenia, and thrombocytopenia, as these are well-recognised adverse effects. In cases of significant neutropenia (ANC < 0.5 × 10^9^/L), treatment should be temporarily discontinued or supported with granulocyte colony-stimulating factors if necessary. Valganciclovir can also, though rarely, cause transaminitis, which is generally mild and self-limiting. Concerns have been raised regarding its cytotoxic properties, as animal studies have shown teratogenic effects and subfertility; however, no such reports have been documented in humans to date (58).

Given undesirable side effects, the role of Therapeutic Drug Monitoring (TDM) for both ganciclovir and valganciclovir has been explored. However, most PK data have been largely derived from adult studies. The adult trough level of 0.5–1.0 mg/L and peak dose level of 7–9 mg/L (taken at one hour post dose for ganciclovir) or 5–7 mg/L (taken at two hours post dose for valganciclovir) have been referenced; however, this has led to suboptimal levels in infants (59–60). The unpredictability of trough and peak levels has led to using Area Under the Curve at 12 hours (AUC0–12h) as a suitable TDM alternative using a paediatric reference range of 20–55 mg × h/L (55). However, the use of TDM in Malaysia is limited and currently unavailable for clinical practice.

### Treatment Availabilities and Alternatives

Despite the established effectiveness of ganciclovir and valganciclovir in treating cCMV, access to these medications remains limited in many regions of Malaysia. Valganciclovir, for example, is only available in tablet form, requiring compounding into a liquid formulation under sterile conditions to extend its shelf life. Its infrequent use has further limited availability to centres specialising in oncology or transplant services. Although generic versions are available in Malaysia, the high cost of treatment remains a major barrier. Another antiviral, foscarnet, which inhibits viral DNA polymerase, is seldom used for cCMV treatment. However, it may have a role in managing cases involving immunocompromised infants or difficult-to-clear CMV from suspected resistant mutations (61).

### Preventive Therapy

Some studies have investigated the potential of treating seropositive mothers as a strategy to prevent transmission to the fetus. One observational study reported that pregnant women treated with valacyclovir had a lower risk of fetal transmission at the time of amniocentesis and showed a good safety profile (62). Valacyclovir appeared more effective in reducing fetal transmission when given during the first and second trimesters (63). A meta-analysis found that prenatal valacyclovir therapy in the first trimester reduces the risk of cCMV infection and increases the likelihood of asymptomatic cCMV infection. Yet, limited evidence exists for established fetal infection (64). Larger studies are necessary before it can be recommended as routine practice.

## Monitoring and Long-Term Management of cCMV

Long-term follow-up is essential for children with cCMV due to the risk of persistent comorbidities, including progressive sensorineural hearing loss (SNHL) and neurodevelopmental delays. Prospective studies have shown SNHL prevalence ranging from 12.7% to 32.7% following cCMV infection (65–66). The risk of progressive SNHL is lower among infants with asymptomatic disease, especially those with normal hearing at birth, a normal cerebral ultrasound, and a normal platelet count (67). However, all children diagnosed with cCMV, regardless of symptoms at birth, should receive regular audiological assessments, as early detection and intervention are critical for optimal speech and language development.

### Audiological Assessments and Monitoring

Audiological assessments of infants with cCMV therefore become an integral component of cCMV management. Universal neonatal hearing screening (UNHS) protocols vary widely in modality and stages. Many utilise a combination of otoacoustic emissions (OAE) and automated auditory brainstem response (AABR) to improve detection and reduce referral rates. However, OAE alone may be used when resources are limited (68–69). Both OAE and AABR demonstrate high sensitivity and specificity. AABR is particularly valuable because it can detect retrocochlear pathologies, including auditory neuropathy spectrum disorder (ANSD) (70–71). Dual-modality and two-stage approaches, especially those combining AABR and OAE, have been associated with better screening outcomes (72). It is important to note that audiology follow-up should continue every six months until at least three to five years of age, as children beyond this period are less likely to develop late-onset SNHL (73). Depending on severity, infants with SNHL should be counselled for early intervention, including hearing aids and potential cochlear implantation.

### Neurodevelopment Assessment and Intervention

The impact of cCMV on neurodevelopment is complex and remains an important area of research. A large cohort study found that nearly 30% of children with cCMV experienced neurodevelopmental impairments, ranging from mild to severe, with symptomatic infants being more frequently affected (74). A scoping review identified several neurological outcomes, including global developmental delay, lower cognitive scores and functioning, speech and language comprehension difficulties, gross motor impairment, hypotonia, and an elevated risk of neurobehavioral conditions such as Autism Spectrum Disorder (ASD) and Attention-Deficit/Hyperactivity Disorder (ADHD) (34). Given these potential complications, regular neurodevelopmental assessments during early childhood are crucial to ensure timely support and intervention, including Early Intervention Programmes (EIP), physiotherapy, occupational therapy, and speech therapy. A multidisciplinary approach involving audiologists, developmental paediatricians, infectious disease specialists, and special education services is vital to optimising long-term outcomes.

## Prospect of cCMV Care: Education, Challenges and Opportunities

### Health Awareness: Professionals and Community

Medical professionals, including paediatricians and neonatologists, demonstrate insufficient knowledge and practice in diagnosing and managing cCMV, with the majority citing inadequate medical training (75). Only 23% of respondents accurately identified the prevalence of cCMV, which is further reflected in the misidentification of the appropriate diagnostic time frame (75). A combined survey of obstetricians and pregnant women revealed limited knowledge in both groups, with obstetricians frequently failing to implement preventive measures due to lack of awareness regarding fetal transmission risks (76). Alarmingly, 31.8% of paediatricians were unaware that newborns with cCMV could be asymptomatic postnatally (77). These gaps in knowledge suggest that a significant number of cCMV cases may go undiagnosed.

Given the absence of a universal cCMV screening consensus, healthcare professionals must improve their understanding of the condition to enable early detection and enhance patient outcomes. Pregnant mothers should be educated on proper hand hygiene practices, especially those at risk with close contact involving young children, either at home or in the workplace, such as childcare settings. Furthermore, promoting health campaigns and encouraging self-reporting by symptomatic mothers could support early screening.

### Early Detection and Intervention

Given the time-sensitive nature of cCMV diagnosis, screening infants at risk has remained a topic of growing interest. The widely implemented universal newborn hearing screening can help identify infants with symptomatic cCMV. However, since most cCMV-infected infants are asymptomatic at birth and serological methods lack sufficient sensitivity, incorporating DBS PCR screening for CMV has been proposed. Universal DBS screening has identified an additional 312–373 cases of cCMV per 100,000 live births, with the potential to reduce long-term healthcare costs associated with non-treatment (78). A Bayesian model using geographically weighted analysis to estimate quality-adjusted life years (QALY) concluded that universal cCMV screening is more cost-effective than targeted screening (79). However, large-scale screening programmes require significant financial investment, including additional assessments such as MRI to detect subclinical but potentially symptomatic infants and increased access to costly antiviral therapies.

**Figure 1:**
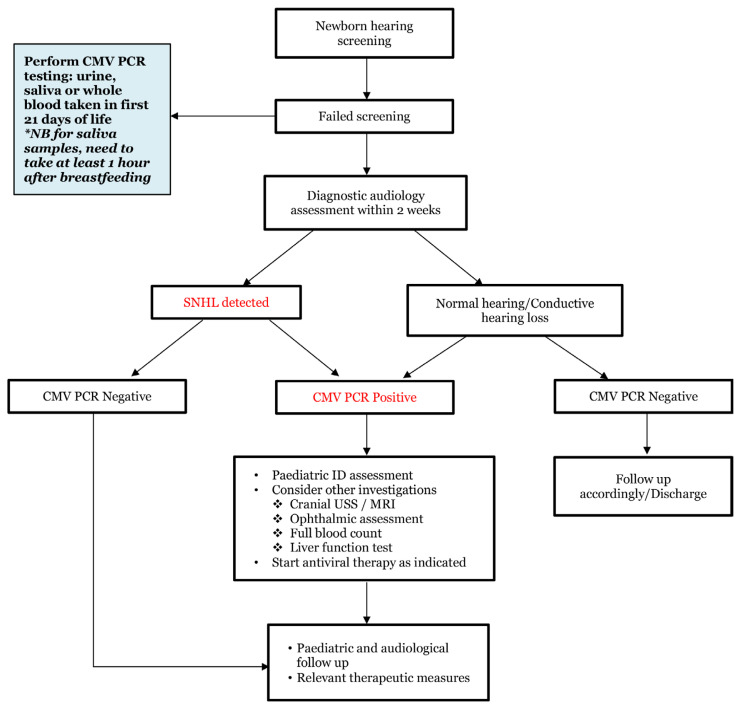
Suggested cCMV referral pathway following failed newborn hearing assessment

In resource-limited settings such as Malaysia, targeted screening provides a cost-effective approach to detecting cCMV among high-risk infants. Infants who fail their initial hearing screening should be offered CMV testing, as one study found that nearly 5% of infants who failed the hearing screen tested positive for CMV infection, higher than the estimated prevalence of cCMV (80). Establishing a referral pathway for infants who fail their hearing screening is crucial to ensure timely evaluations and appropriate treatment (Figure 1). However, relying solely on UNHS for targeted cCMV detection has limitations, as approximately 43% of CMV-positive infants with late-onset sensorineural hearing loss (SNHL) were not identified at birth (81). This highlights the progressive nature of audiological complications and underscores that a negative newborn hearing screen does not reliably exclude cCMV infection in at-risk infants.

A scoring system that integrates maternal and neonatal risk factors could improve the sensitivity of CMV detection. However, no validated system currently exists. Establishing one would require a large dataset to assess correlations between maternal and neonatal risk factors with symptom severity, assisting in the identification of parameters that predict complications such as SNHL. A national or regional collaborative registry, such as the Congenital CMV Network (CCMVNET), could provide essential data to enhance understanding of the disease’s full spectrum and impact (82). Machine learning modelling based on these data could serve as a sophisticated screening tool for cCMV diagnosis.

Nonetheless, a simplified “Likelihood of diagnosis” table incorporating clinical and investigative features is proposed (Table 1).

Infants categorised as “Suggestive” and “Possible” should be offered a CMV qPCR confirmatory test. In contrast, those classified as “To rule out” should be tested if no other explanation for their abnormal features is identified. Since the decision to treat is equally challenging and access to antivirals is limited, establishing a clinical framework is essential for categorising infants who would benefit from therapy along with the suggested duration (Table 2).

### Expanding Diagnostic Tests

Current confirmatory diagnostic methods for cCMV rely on detecting CMV DNA in saliva, urine, or DBS using qPCR. Although these methods are highly sensitive and specific, they require considerable resources, including specialised laboratory infrastructure and skilled personnel. These limitations render qPCR-based testing less practical, particularly in resource-limited settings such as Malaysia, where molecular testing is often centralised. Identifying alternative biomarkers, such as CMV-specific antigens, could permit the development of rapid, affordable, and decentralised point-of-care tests. Phosphoprotein 65 (pp65) antigen, for example, are abundantly expressed during active CMV replication and have been successfully employed in other clinical contexts, such as antigenemia assays for immunocompromised patients (83). However, their potential for cCMV diagnosis remains unexplored. Investigating these antigens could support the development of rapid diagnostic tests (RDTs) using lateral flow immunoassays or nanobiosensors that could transform existing screening methods.

### Vaccine Advancements

No CMV vaccines have been approved, although significant progress has been achieved in vaccine development. Researchers have still to determine an optimal target for this highly complex virus, which encodes over 200 proteins and has sophisticated immune evasion mechanisms, but several vaccine platforms have been proposed (84). Nonetheless, a preliminary study on adults demonstrated a promising vaccine candidate that was safe, well-tolerated, and capable of eliciting a desirable antigen-specific immune response (85). Research efforts have primarily focused on vaccinating women of childbearing age to prevent maternal-to-fetal transmission. While these advancements are encouraging, additional research and the successful completion of ongoing clinical trials are essential before a CMV vaccine can become broadly available.

## Conclusion

Congenital CMV remains a significant yet underrecognised public health concern in Malaysia, with limited screening, diagnostic accessibility, and awareness among healthcare providers. Advances in molecular testing, targeted screening strategies, and emerging biomarkers provide potential for earlier and more cost-effective detection. Improved access to antiviral treatment and long-term follow-up is vital to mitigating neurodevelopmental and audiological complications. Future efforts should focus on integrating cCMV management into maternal and child health programmes, expanding research on novel diagnostics, and enhancing public and professional awareness. Strengthening national policies and fostering international collaborations will be pivotal in reducing the burden of cCMV.

## Figures and Tables

**Table 1:** Likelihood of cCMV diagnosis

Likelihood of cCMV	Clinical features/investigations	Findings
Diagnostic In a sample taken from infants less than or equal to 21 days old	CMV molecular test, ie. PCR (qualitative/quantitative)	CMV detected
	Fetal amniotic fluid	
	Infant urine (preferred)	
	Infant saliva (collect sample one hour after breastfeeding to avoid detecting CMV secreted in breast milk)	
	*Infant Whole blood (false negative result may occur)	

Suggestive	Maternal CMV IgM and IgG (with avidity) AND one or more of the symptomatic clinical features	Positive IgM/IgG with low IgG avidity
		Small for gestational age
		Prematurity
		Microcephaly
		Hepatosplenomegaly
		Jaundice (prolonged/conjugated)
		Skin manifestations such as blueberry muffin
		rash, petechiae or purpura
		Hypotonia, seizures

	AND/OR	Anaemia
	one or more unexplained haematological or biochemical features	Neutropenia
		Thrombocytopenia Transaminitis

	AND/OR	Cranial USS:
	one or more radiological features	LSV, periventricular calcifications, cysts, and ventriculomegaly
		MRI brain:
		Multifocal white matter lesions, periventricular cysts, cortical malformations including polymicrogyria and lissencephaly, cerebellar hypoplasia
	AND / OR	Failed or no response
	Audiology assessment (OAE/AABR)	*Diagnostic ABR recommended

	AND / OR	Chorioretinitis, strabismus, amblyopia
	Ophthalmology assessment	Congenital cataracts

Possible	Maternal CMV IgM and IgG (with avidity)	Positive IgM/IgG with low IgG avidity
	AND	
	Absence of symptoms and normal investigations	

To rule out	Maternal CMV IgM and IgG (with avidity)	Unknown maternal serology
	AND	OR
	At least one or more of the clinical, haematological, biochemical, radiological, audiological, or ophthalmological features	Positive IgM/IgG with high or unknown IgG avidity

Discarded	CMV molecular test, ie. PCR (qualitative/quantitative) – urine, saliva, amniotic fluid	CMV not detected

Infants identified as “suggestive/possible” should undergo urgent confirmatory PCR CMV testing, while “to rule out” should be tested if no other causes are found; AABR = automated auditory brainstem response; ABR = auditory brainstem response; CMV = cytomegalovirus; LSV = lenticulostriate vasculopathy; MRI = Magnetic Resonance Imaging; OAE = otoacoustic emissions; PCR = polymerase chain reaction; USS = ultrasound scan

**Table 2:** Which infant to treat and how long?

Severity of cCMV disease	Clinical features	Treatment decision and duration of valganciclovir
Asymptomatic	-	No treatment required

Mild/moderate	Isolated thrombocytopenia	No treatment is usually needed as disease often transient and self-limiting
	Isolated anaemia	
	Isolated neutropenia	Consider six weeks course if: persistent symptoms (> 2 weeks); or two or more of these symptoms
	Isolated hyperbilirubinaemia or transaminitis	
	Isolated small for gestational age	
	Isolated petechiae	
	Isolated mild hepatosplenomegaly	

Severe	Severe single-organ or severe multiple-organ disease (without CNS involvement or hearing impairment)	Start six weeks course
	Sensorineural deafness (unilateral/bilateral)	Start six months course
	Evidence of CNS disease including microcephaly or any suggestive intracranial findings on USS/MRI	
	Chorioretinitis	

Adapted from Luck et al. (25); CNS = central nervous system; MRI = Magnetic Resonance Imaging; USS = ultrasound scan
